# Green tea extract suppresses adiposity and affects the expression of lipid metabolism genes in diet-induced obese zebrafish

**DOI:** 10.1186/1743-7075-9-73

**Published:** 2012-08-07

**Authors:** Takahiro Hasumura, Yasuhito Shimada, Junya Kuroyanagi, Yuhei Nishimura, Shinichi Meguro, Yoshinori Takema, Toshio Tanaka

**Affiliations:** 1 Biological Science Laboratories, Kao Corporation, 2606 Akabane, Ichikai-machi, Haga-gun, Tochigi, 321-3497, Japan; 2 Department of Molecular and Cellular Pharmacology, Pharmacogenomics and Pharmacoinformatics, Mie University Graduate School of Medicine, 2-174 Edobashi, Tsu, Mie, 514-8507, Japan; 3 Department of Bioinformatics, Mie University Life Science Research Center, 2-174 Edobashi, Tsu, Mie, 514-8507, Japan; 4 Department of Omics Medicine, Mie University Industrial Technology Innovation Institute, 2-174 Edobashi, Tsu, Mie, 514-8507, Japan; 5 Mie University Medical Zebrafish Research Center, 2-174 Edobashi, Tsu, Mie, 514-8507, Japan; 6 Research and Development, Kao Corporation, 2-1-3 Bunka, Sumida-ku, Tokyo, 131-8501, Japan

**Keywords:** Body fat, Catechin, Diet-induced obesity, 3D micro-computed tomography, Green tea extract, SOCS3, Zebrafish

## Abstract

**Background:**

Visceral fat accumulation is one of the most important predictors of mortality in obese populations. Administration of green tea extract (GTE) can reduce body fat and reduce the risk of obesity-related diseases in mammals. In this study, we investigated the effects and mechanisms of GTE on adiposity in diet-induced obese (DIO) zebrafish.

**Methods:**

Zebrafish at 3.5 to 4.5 months post-fertilization were allocated to four groups: non-DIO, DIO, DIO + 0.0025%GTE, and DIO + 0.0050%GTE. The non-DIO group was fed freshly hatched *Artemia* once daily (5 mg cysts/fish daily) for 40 days. Zebrafish in the three DIO groups were fed freshly hatched *Artemia* three times daily (60 mg cysts/fish daily). Zebrafish in the DIO + 0.0025%GTE and DIO + 0.0050%GTE groups were exposed to GTE after the start of feeding three times daily for 40 days.

**Results:**

Three-dimensional microcomputed tomography analysis showed that GTE exposure significantly decreased the volume of visceral but not subcutaneous fat tissue in DIO zebrafish. GTE exposure increased hepatic expression of the lipid catabolism genes *ACOX1* (*acyl-coenzyme A oxidase 1, palmitoyl*), *ACADM* (*acyl-coenzyme A dehydrogenase, c-4 to c-12 straight chain)*, and *PPARA* (*peroxisome proliferator-activated receptor alpha*). GTE exposure also significantly decreased the visceral fat expression of *SOCS3* (*suppressor of cytokine signaling 3b*) which inhibits leptin signaling.

**Conclusions:**

The present results are consistent with those seen in mammals treated with GTE, supporting the validity of studying the effects of GTE in DIO zebrafish. Our results suggest that GTE exerts beneficial effects on adiposity, possibly by altering the expression of lipid catabolism genes and *SOCS3*.

## Background

According to 2005 World Health Organization estimates, approximately 1.6 billion adults worldwide were overweight (body mass index [BMI] ≥ 25 kg/m^2^) and at least 400 million were obese (BMI ≥ 30 kg/m^2^), and these numbers are expected to reach 2.3 billion and 700 million, respectively, by 2015 [1]. It is widely accepted that obesity results from a positive energy balance [2]. In general, obesity can be classified into visceral and subcutaneous types according to the distribution of fat. Visceral obesity accompanies or precedes components of the metabolic syndrome, such as hyperinsulinemia and insulin resistance [3]. Consequently, visceral fat accumulation is an independent predictor of mortality in men [4].

Green tea, one of the most popular beverages in Asian countries, contains numerous polyphenols known as catechins, particularly epigallocatechin gallate, epicatechin gallate, and gallocatechin gallate. Tea and tea components have many beneficial properties, including antioxidant [5], anticancer [6,7], antidiabetic [8], and anti-atherogenic [9] activities. Moreover, intake of tea catechins inhibits diet-induced obesity (DIO) in mice [10,11] and reduces body weight and body fat in humans [12-17]. These anti-obesity effects of tea catechins seem to involve stimulation of fat oxidation [10,18,19], modulation of adipogenesis [20], decreased fat synthesis [6,20-22], and inhibition of digestive enzyme activity and nutrient absorption [8].

Zebrafish (*Danio rerio*) are vertebrates, and their organs and tissues show similarities to those of humans in terms of their structure and function. Consequently, zebrafish are increasingly being used as models of human diseases [12,23-25] because of their ease of genetic manipulation and their economic potential for use in breeding and testing. In addition, lipid metabolism in zebrafish is very similar to that in humans in terms of intestinal absorption with the aid of bile produced in the liver [26], transport of fat and cholesterol by lipoproteins [27], β-oxidation [28], and storage as triacylglycerols in visceral, subcutaneous, and intramuscular adipocyte depots [26,29]. Because of these similarities and advantages, zebrafish are used in lipid metabolism research as a model for lipid-related diseases, including atherosclerosis induced by high-cholesterol diets [30] and obesity induced by overexpression of the endogenous melanocortin antagonist agouti-related protein (AgRP) [29]. Moreover, Oka et al. demonstrated the usefulness of a zebrafish model of DIO that shares common pathophysiological pathways with mammalian obesity [31]. DIO zebrafish have also been used to validate the anti-obesity effects of natural products [32].

Here, we investigated the effects of green tea extract (GTE) on body weight, visceral and subcutaneous fat accumulation, and the expression of lipid metabolism genes in the liver and visceral fat in DIO zebrafish.

## Methods

### Ethical approval

This study conformed to the ethical guidelines established by the Institutional Animal Care and Use Committee of Mie University.

### GTE

GTE was prepared and analyzed as previously described [33]. In brief, green tea leaves (*Camellia sinensis*) were soaked in hot water and the resulting extract was reduced to a powder by spray drying. The extract was then dissolved in hot water and mixed with an equal volume of chloroform. The aqueous phase was recovered with three volumes of ethanol and the extract was freeze-dried after removing the solvent. The composition of catechins was measured by high-performance liquid chromatography. The total catechin content in the GTE was 79.6%–81.8% (w/w, the sum of all catechins); this comprised epigallocatechin gallate (43.6%–44.4%), epigallocatechin (20.4%–20.7%), epicatechin gallate (12.0%–12.3%), epicatechin (8.1%–8.3%), gallocatechin (6.9%–7.0%), gallocatechin gallate (4.4%), and others (3.7%–3.8%). The caffeine content was 0%–0.1%.

### Animals

Male and female zebrafish (AB strain, the Zebrafish International Resource Center, Eugene, OR, USA) were kept at approximately 28°C under a 14-h light and 10-h dark cycle. Water conditions of environmental quality were maintained according to *The Zebrafish Book *[34].

### Experimental design

#### Experiment 1

Male and female zebrafish at 3.5–4.5 months post-fertilization were allocated to four groups (non-DIO, DIO, DIO + 0.0025%GTE, and DIO + 0.0050%GTE) with five fish per 1.7-L tank. Zebrafish in the non-DIO (control group) were fed freshly hatched *Artemia* for 120 min once a day (5 mg cysts/fish daily) for 40 days. Zebrafish in the three DIO groups were fed freshly hatched *Artemia* for 120 min three times daily (60 mg cysts/fish daily). Zebrafish in the DIO + 0.0025%GTE and DIO + 0.0050%GTE groups were exposed to GTE for 105 min, starting 15 min after the start of feeding (total exposure, 315 min/day), three times daily for 40 days. During feeding, the tank water flow was stopped; the water in each tank was replaced with fresh water at the end of feeding. Body weight was measured on days 0, 14, 20, 27, 34, and 40. Body fat volume was measured using three-dimensional microcomputed tomography (3D micro-CT) following euthanasia on the final day of the study.

#### Experiment 2

Female zebrafish were allocated to three groups (non-DIO, DIO, and DIO + 0.0050%GTE) with five fish per 1.7-L tank. All groups were fed as described in *Experiment 1* for 21 days. After feeding on the final day, the zebrafish were euthanized, immediately transferred into tubes containing 8 mL of RNAlater (Qiagen, Valencia, CA, USA), and stored at 4°C until gene expression analysis.

### CT measurement of body fat volume

**Figure 1:**
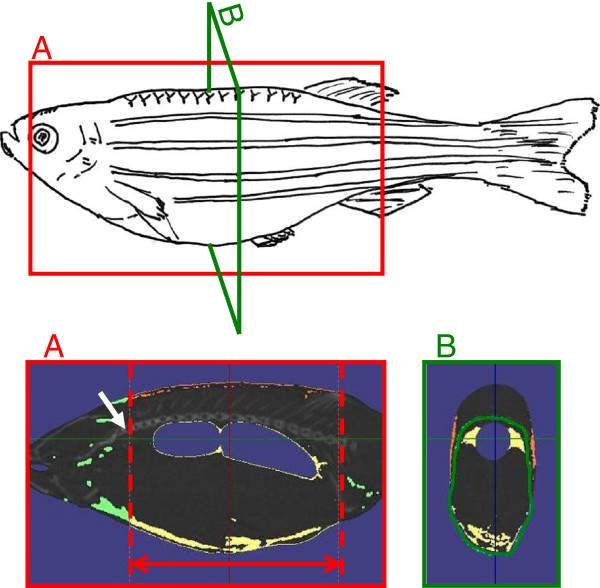
**Cross-sectional images taken by three-dimensional micro-computed tomography. **The diagram of the zebrafish shows where the two cross-sectional images A and B were taken. (**A**) The red two-headed arrow shows the area where body fat volume was measured (yellow). (**B**) The green line shows the boundary between the visceral fat and the subcutaneous fat (orange). The light green area indicates body fat outside the measuring area. The white arrow indicates the cleithrum.

Zebrafish were fixed in a stretched position on a sample holder. The 3D micro-CT scan was performed with an *in vivo* System R_mCT 3D micro-CT scanner (Rigaku Corporation, Tokyo, Japan). The following settings were used: voltage, 90 kV; current, 100 μA; magnification, ×4; slice thickness (scanning width), 50 μm; and exposure time, 2 min. Images were reconstructed and viewed using i-View type R software (J. Morita Mfg., Kyoto, Japan). The CT images were visualized and analyzed using CTAtlas Metabolic Analysis Ver. 2.03 software (Rigaku Corporation). The Hounsfield unit (HU) value of fat tissue was adjusted to between –350.0 and –145.0 in accordance with the manufacturer’s instructions. Measurement of body fat volume was limited to the abdominal cavity, and the initial point of the abdominal cavity was set at the cleithrum (Figure 1A). Body fat was then divided into visceral fat and subcutaneous fat along the ribs (Figure 1B).

### RNA extraction and quantitative real-time PCR

The liver and visceral fat were dissected from the stored fish samples prepared in *Experiment 2* and were subjected to RNA extraction. Total RNA was extracted from the livers of fish in all three groups (non-DIO, DIO, and DIO + 0.0050%GTE) using Isogen (Nippongene, Tokyo, Japan) with combination with cleanup protocol of RNeasy mini kit (Qiagen K.K., Tokyo, Japan). cDNA was synthesized using random primers and SuperScript III Reverse Transcriptase (Invitrogen, Carlsbad, CA, USA). Total RNA was extracted from the visceral fat of fish in the DIO and DIO + 0.0050%GTE groups using an RNeasy Lipid tissue mini kit (Qiagen) and qualified using an Agilent Bioanalyzer 2100 (Agilent, Santa Clara, CA, USA). Because of poor RNA quality, the samples for two of five fish in the DIO group were excluded. cDNA was synthesized using a High Capacity RNA-to-cDNA Kit (Applied Biosystems, Foster City, CA, USA). Total RNA could not be extracted from the visceral fat of fish in the non-DIO group because the amount of visceral fat was too small.

Quantitative real-time PCR was performed on cDNA samples using a TaqMan Fast Universal PCR Master Mix (Applied Biosystems) or Fast SYBR Green Master Mix (Applied Biosystems) and an ABI Prism 7500 Fast Real-Time PCR System (Applied Biosystems) in accordance with the manufacturer’s instructions. The TaqMan gene expression assays were as follows: *PPIA* (*peptidylprolyl isomerase Aa*; Dr03152038_m1), *ACOX1* (*acyl-coenzyme A oxidase 1, palmitoyl*; Dr03147239_m1), *ACADM* (Dr03120754_m1), *PPARA* (*peroxisome proliferator-activated receptor alpha b*; Dr03149883_m1), and *SOCS3* (*suppressor of cytokine signaling 3b*; Dr03203997_s1). The primer sets for SYBR Green were as follows: *ACC* (*acetyl-CoA carboxylase* gene, XM_678989, forward primer (5′–3′): ATCATCCCACCCAAACAGAC; reverse primer (3′–5′): CCCATCACAGAAGGTGGAAC) and *FASN* (*fatty acid synthase* gene, XM_682295, forward primer (5′–3′): ATCTGTTCCTGTTCGATGGC, reverse primer (3′–5′): AGCATATCTCGGCTGACGTT). The baseline and threshold were set manually in accordance with the manufacturer’s instructions. The relative mRNA expression levels were determined using *PPIA* as an endogenous standard.

### Feeding volume assay

The feeding volume assay was conducted as previously described [32], with minor modifications, on day 39 of *Experiment 1*. Briefly, hatched *Artemia* (5 or 60 mg cysts/fish/day) were fed to the zebrafish in a 1.7-L tank as described above. For a blank control, *Artemia* were placed in a 1.7-L tank without zebrafish (the tank contained water alone). After 90 min, the number of *Artemia* not eaten by the zebrafish were counted three times and subtracted from the number in the blank tank to determine the feeding volume in each tank.

### Statistical analysis

All values are presented as means ± standard error of the mean. Statistical analysis was conducted using analysis of variance followed by Fisher’s partial least-squares difference multiple comparison test. Analyses were conducted using STATVIEW for Windows version 5.0 (SAS Institute Inc., Cary, NC, USA). Values of *P* < 0.05 were considered statistically significant.

## Results

### Food intake

Visual observation revealed no marked abnormalities or major differences in feeding behavior between the three DIO groups (i.e., DIO, DIO + 0.0025%GTE, and DIO + 0.0050%GTE). The ratio of *Artemia* consumed to the amount of *Artemia* provided (consumption ratio) was estimated in the feeding volume assay. In male zebrafish, the consumption ratio in the DIO + 0.0025%GTE (40%) and DIO + 0.0050%GTE (37%) did not decrease compared with that in the DIO group (24%). And also in female, the consumption ratio in the DIO + 0.0025%GTE (33%) and DIO + 0.0050%GTE (38%) did not decrease compared with that in the DIO group (33%).

### Body weight

**Figure 2:**
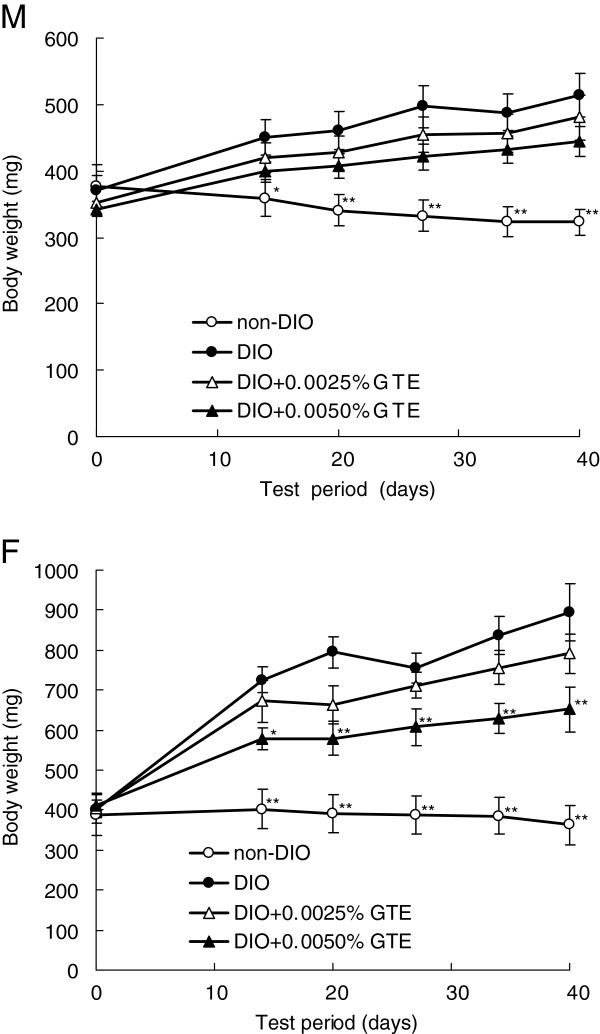
**Effects of green tea extract (GTE) on body weight. **Changes in body weight in male (**M**) and female (**F**) zebrafish. Values are means ± SE. **P < 0.01 and *P < 0.05 vs. the diet-induced obese (DIO) group.

Significant increases in body weight were observed within 14 days of the start of the experiment in male and female zebrafish in the DIO group compared with those in the non-DIO group (Figure 2). This trend was maintained throughout the 40-day study. From day 14 onward, exposure to 0.0050% GTE significantly reduced the diet-induced body weight gain in females (*P* < 0.05 or *P* < 0.01 compared with the DIO group). By contrast, there were no significant reductions in diet-induced body weight gain in males exposed to either dose of GTE, or in females exposed to 0.0025% GTE.

### Body fat volume

**Figure 3:**
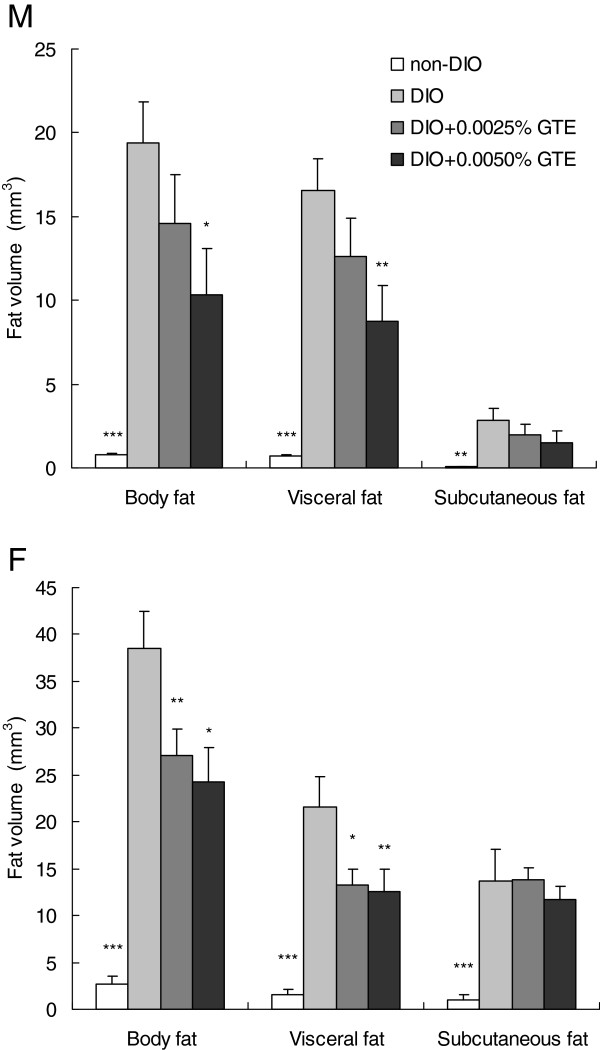
**Effects of green tea extract (GTE) on body fat volume. **Mean body fat volume in male (**M**) and female (**F**) zebrafish after 40 days of feeding. Values are means ± SE. ***P < 0.001, **P < 0.01, and *P < 0.05 vs. the diet-induced obese (DIO) group.

To quantify body fat volume in DIO zebrafish, we performed 3D micro-CT analysis. This allowed us to quantify the total body fat volume and to separately quantify the visceral and subcutaneous fat volumes. Figure 3 compares the body fat volumes between each experimental group. Total body fat, visceral fat, and subcutaneous fat volumes in both sexes in the DIO group were significantly greater than those in the non-DIO group. Total body fat and visceral fat volumes in male DIO zebrafish were significantly reduced by exposure to 0.0050% GTE, and those in female DIO zebrafish were significantly reduced by both doses of GTE. However, in both sexes, there were no significant differences in subcutaneous fat volume between the DIO group and either GTE-treated group.

### Liver and visceral fat mRNA expression in females

We analyzed the mRNA expression levels of lipid metabolism genes in the liver and visceral fat tissue of female DIO zebrafish because the suppressive effects of GTE on body fat accumulation were greater in females than in males.

**Figure 4:**
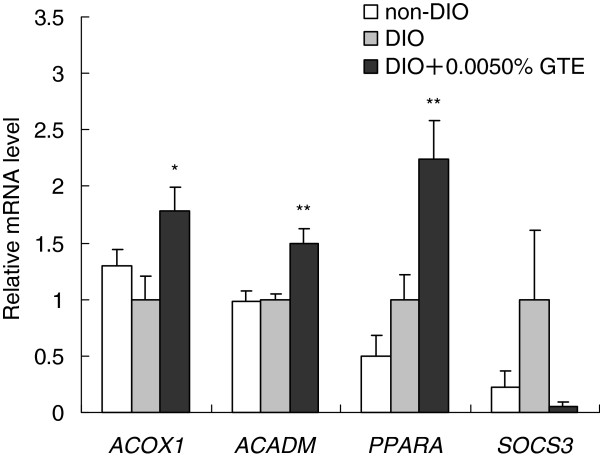
**Effects of green tea extract (GTE) on the expression of lipid metabolism genes in the liver. ***ACOX1*, *ACADM*, *PPARA*, and *SOCS3* mRNA expression in the liver of female zebrafish on day 21 of feeding. The expression of each mRNA was normalized against *PPIA* mRNA expression and the corresponding expression in the diet-induced obese (DIO) group. Values are means ± SE. **P < 0.01 and *P < 0.05 vs. the DIO group.

In the liver on day 21 of the experiment, the mRNA expression levels of the mRNAs encoding *ACOX1* (a peroxisomal β-oxidation enzyme), *ACADM* (a mitochondrial β-oxidation enzyme), and *PPARA* (a nuclear receptor protein that regulates β-oxidation) were significantly upregulated in the DIO + 0.0050%GTE group compared with the DIO group (Figure 4). By contrast, there was no significant difference in the mRNA expression of *SOCS3* (a protein that suppresses leptin signaling).

**Figure 5:**
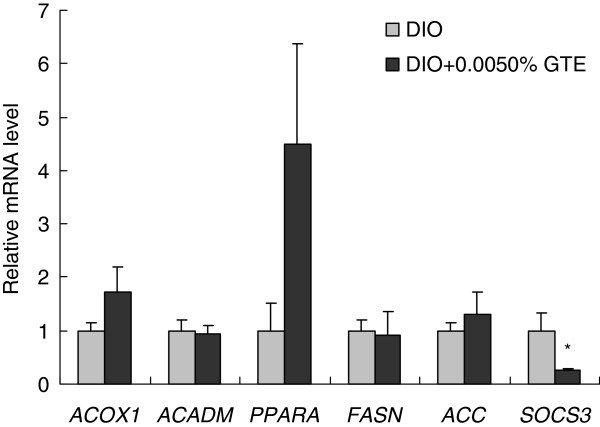
**Effects of green tea extract (GTE) on the expression of lipid metabolism genes in visceral fat. ***ACOX1*, *ACADM*, *PPARA*, *FASN*, *ACC*, and *SOCS3* mRNA expression in the visceral fat of female zebrafish on day 21 of feeding. The expression of each mRNA was normalized against *PPIA* mRNA expression and the corresponding expression in the diet-induced obese (DIO) group. Values are means ± SE. *P < 0.05 vs. the DIO group.

In the visceral fat on day 21 of the experiment, the mRNA expression levels of *ACOX1*, *ACADM*, and *PPARA* in the DIO + 0.0050%GTE group were not significantly differ from those in the DIO group. In addition, there were no significant differences in the mRNA expression levels of *FASN* (*fatty acid synthase*) or *ACC* (*acetyl-CoA carboxylase*), which play important roles in lipogenesis. On the other hand, the mRNA expression of *SOCS3* was significantly lower in the DIO + 0.0050%GTE group than in the DIO group (Figure 5).

## Discussion

In this study, we demonstrated that GTE significantly suppressed the accumulation of visceral fat, but not subcutaneous, in a concentration-dependent manner in zebrafish. This effect was accompanied by increase hepatic expression of lipid catabolism genes. These results are very consistent with the findings of previous studies in humans [12,14] and rodents [10,11,13,14,35-37]. Although it is unclear whether the functions of visceral and subcutaneous fat in zebrafish are homologous to those in mammals, our findings indicate that body fat distribution is present in zebrafish as well as in humans and animals and that the site-specific effects of GTE are similar to those in mammals.

The mechanisms underlying the suppressive effects of GTE on body fat accumulation have been investigated in rodent models. Some studies have shown that upregulated PPARs and many lipid-metabolizing enzymes stimulate fat oxidation in the liver [11,35,36]. The hepatic gene expression pattern observed in DIO zebrafish in our study support these earlier findings. Although it was recently reported that the expression of lipogenic genes, such as *FASN*, is downregulated in visceral fat tissue in obese mice [38], similar changes were not observed in our study. We assume that the reason for this discrepancy is the difference in obesity models used. In particular, Park et al. used obese mutant mice with increased *FASN* gene expression in adipose tissue [38], whereas we used a wild-type strain of fish. In support of the hypothesis that the activities of FASN differs between models of obesity, Letexier et al. reported that adipose tissue *FASN* expression is not affected by a high-fat diet in wild-type rodents and is reduced in obese humans [39]. Their results help support the notion that adipose tissue gene expression profiles differ among species, and the lack of consistency between our data and those of Park et al. Nevertheless, our present results suggest that the mechanism by which GTE suppresses body fat accumulation, at least in the liver, in zebrafish is similar to that in rodents.

We also found that GTE significantly decreased the expression of *SOCS3* in the visceral fat of DIO zebrafish. SOCS3 is a negative regulator of leptin signaling and was recently proposed as an important therapeutic target for obesity [40]. SOCS3 production in fat is associated with obesity in humans and rodents as *SOCS3* gene expression is increased in subcutaneous fat of obese patients [41], and its protein and gene expression levels are increased in epididymal fat of DIO rodents [42,43]. We found that GTE suppressed adipose tissue *SOCS3* expression in zebrafish, which was accompanied by reduced body fat accumulation. To our knowledge, these results suggest for the first time that *SOCS3* expression is correlated with body fat volume in zebrafish and is involved in the regulation of body fat volume by GTE.

Another valuable outcome of this study is that 3D micro-CT analysis can be used to analyze the effect of compounds on adiposity in DIO zebrafish. In humans, visceral fat accumulation, unlike subcutaneous fat accumulation, is strongly associated with metabolic and vascular risks, particularly diabetes, hypertension, and stroke, and thus increases the risk of mortality [3]. CT is widely used to measure body fat volume in humans [44,45] and in rodents [46,47]. Although CT is used to evaluate fillet composition in teleosts, such as salmon and carp [48-50], to our knowledge, no reports have used CT to measure body fat volume or visceral/subcutaneous fat volume in small teleosts. Our data raise the possibility that micro-CT could be used to measure body fat volume in such fish. We believe this technique will play an important role in future studies of lipid metabolism and obesity in zebrafish.

## Conclusions

We showed that GTE significantly inhibits weight gain and body fat accumulation, and alters the expression of hepatic lipid catabolism genes in DIO zebrafish. Our results suggest that GTE exerts beneficial effects on adiposity, possibly by altering the expression of lipid catabolism genes and *SOCS3*.

## Abbreviations

*ACADM*: *Acyl-coenzyme A dehydrogenase, c-4 to c-12 straight chain* gene; *ACC*: *Acetyl-CoA carboxylase* gene; *ACOX1*: *Acyl-coenzyme A oxidase 1, palmitoyl* gene; AgRP: Agouti-related protein; BMI: Body mass index; CT: Computed tomography; DIO: Diet-induced obesity; *FASN*: *Fatty acid synthase* gene; GTE: Green tea extract; *PPARA*: *Peroxisome proliferator-activated receptor alpha b* gene; *SOCS3*: *Suppressor of cytokine signaling 3b* gene.

## Competing interests

The authors declare that they have no competing interests.

## Authors’ contributions

TH and YS carried out all animal studies. TH drafted the manuscript. JK helped to perform the experiments. YS, NY, MS, TY, and TT conceived the study, participated in its design and coordination, and helped to draft the manuscript. All authors read and approved the final manuscript.

## Authors’ information

Takahiro Hasumura is a researcher in the research and development section of Kao Corporation. The focus of Mr. Hasumura’s research is health science.

Yasuhito Shimada, MD is an assistant professor at the Department of Molecular and Cellular Pharmacology, Pharmacogenomics and Pharmacoinformatics, Graduate School of Medicine, Mie University. The focus of Dr. Shimada’s research is the prevention of obesity using zebrafish as a model of diet-induced obesity.

Yuhei Nishimura, MD, PhD is a lecturer in the Department of Molecular and Cellular Pharmacology, Pharmacogenomics and Pharmacoinformatics, Graduate School of Medicine, Mie University. Dr Nishimura’s research interests are bioinformatics and pharmacoinformatics.

Toshio Tanaka, MD, PhD is a professor in the Department of Molecular and Cellular Pharmacology, Pharmacogenomics and Pharmacoinformatics, Graduate School of Medicine, Mie University. The focus of Dr Tanaka’s research is the identification of drug targets using pharmacogenomic methods. Dr. Tanaka has published more than 100 peer-reviewed research articles and reviews in international journals.
